# High Vulnerability of Oligodendrocytes to Oxidative Stress Induced by Ultrafine Urban Particles

**DOI:** 10.3390/antiox10010004

**Published:** 2020-12-22

**Authors:** Ji Young Kim, Jin-Hee Kim, Yong-Dae Kim, Je Hoon Seo

**Affiliations:** 1 Department of Anatomy, Chungbuk National University College of Medicine, Cheongju 28644, Korea; kjyblue84@gmail.com; 2 Department of Biomedical Laboratory Science, College of Health Science, Cheongju University, Cheongju 28503, Korea; jinheekim23273@gmail.com; 3 Department of Preventive Medicine, Chungbuk National University College of Medicine, Cheongju 28644, Korea; 4 Chungbuk Regional Cancer Center, Chungbuk National University Hospital, Cheongju 28644, Korea

**Keywords:** particulate matter, particle, air pollution, oligodendrocyte precursor cell, mature oligodendrocyte, demyelination, multiple sclerosis

## Abstract

Oligodendrocytes, myelin-forming cells in the brain, are vulnerable to oxidative stress. Recent work indicates that air pollution causes demyelinating diseases such as multiple sclerosis. However, little is known about the mechanism of toxicity of ultrafine particulate matters (PMs) to oligodendrocytes. Here, we aimed to determine whether oligodendrocyte precursor cells (OPCs) and mature oligodendrocytes (mOLs) are more vulnerable to ultrafine urban PMs (uf-UPs) than other types of brain cells and damage to adult OPCs and mOLs in the mouse brain exposed to uf-UPs. For in vitro experiments, following exposure to various concentrations (2, 20, and 200 μg/mL) of uf-UPs, we measured survival rates, the amount of reactive oxygen species (ROS), and the total antioxidant capacities (TACs) of brain cells isolated from neonatal Sprague-Dawley rats. For animal experiments, after a four-week exposure to a uf-UP suspension (20 μL, 0.4 mg/mL), we enumerated the number of damaged cells and typed damaged cells in the white matter of the cerebellum of uf-UP-exposed mice. MTT assays and Hoechst staining demonstrated that OPCs and mOLs were more vulnerable to uf-UP-induced damage than astrocytes and cortical neurons at 2, 20, and 200 μg/mL of uf-UPs examined in this study (*p* < 0.05). Damage to OPCs and mOLs depended on uf-UP concentration. DCF assays and DHE staining indicated that the amount of ROS generated in OPCs and mOLs was significantly higher than in other brain cell types (*p* < 0.05). In contrast, TAC values in OPCs and mOLs were significantly lower than those of other brain cell types (*p* < 0.05). Fluoro-Jade B (FJB)-positive cells in the cerebellar white matter of the uf-UP-exposed group were significantly greater in number relative to the control group. Double immunofluorescence indicated that FJB-positive cells are NG2-positive adult OPCs and carbon anhydrase II-positive mOLs. Taken together, our findings suggest that oxidative stress induced by uf-UPs in the brain impairs adult OPCs and mOLs, causing demyelination and reducing the capacity for remyelination.

## 1. Introduction

Ultrafine particulate matters (PMs) are commonly defined as air pollution particles smaller than 0.2 μm in diameter. Ultrafine PMs have a significantly greater impact on humans than coarse (PM10) and fine PMs (PM2.5), which have larger particle sizes. Unlike coarse and fine PMs, airborne ultrafine PMs can reach the lungs, since they are not filtered by the nose, trachea, or bronchi during normal breathing [1]. In addition, recent studies have shown that the accumulation of ultrafine PMs in alveoli increases the risk of pneumonia and lung cancer [2,3]. Worse, ultrafine PMs can pass through the blood–air barrier of the lung and enter the bloodstream, which can lead to various cardiovascular diseases [4,5,6]. Furthermore, since it has been shown that ultrafine PMs pass through the blood-brain barrier of 14-week-old Fisher-344 rats, it has been argued that they may also be linked to human brain diseases [1].

Experimental evidence has indicated that ultrafine PMs in the brain may increase reactive oxygen species (ROS) production and subject brain cells to damage induced by oxidative stress, leading to Parkinson’s disease and Alzheimer’s disease [7]. Recent cohort studies have demonstrated a strong correlation between air pollution and the risk of multiple sclerosis (MS) caused by demyelination of white matter. Moreover, severe air pollution in Tehran, Iran was found to have caused a surge in the number of MS patients [8]. Another study in Lombardy showed a statistically significant correlation between PM concentration and MS-related hospitalization [9]. These previous findings suggest that ultrafine PMs directly affect both myelin and oligodendrocytes (OLs), myelin-forming cells in the central nervous system. However, to date there have been no empirical studies of this relationship using experimental animals and little is known about the underlying mechanism by which ultrafine PMs damage OLs and cause MS.

Depending on the degree of differentiation, OLs are generally classified as oligodendrocyte progenitor cells (OPCs), premyelinating OLs, or mature myelinating OLs (mOLs). Each type of OL lineage cell has a unique shape and phenotypical antigenicity [10]. In the adult brain, the majority of OL lineage cells are mOLs, although a few adult OPCs remain. Both types of cell play crucial roles in remyelination [11,12]. Since OL lineage cells have lower concentrations of antioxidant enzymes—e.g., glutathione peroxidase and catalase—than other types of brain cell, they are highly vulnerable to oxidative stress [13]. In this study, we evaluated the effect of exposure to ultrafine PMs on OPCs and mOLs via both in vitro and in vivo experiments. We determined whether exposure to ultrafine urban particulate matters (uf-UPs) as ultrafine PMs produced ROS and damaged brain cells isolated from neonate rats. We also examined extent of the damage to cerebellar white matter OPCs and mOLs of Balb/c mice after four weeks of exposure to uf-UPs by nasal instillation.

## 2. Materials and Methods

### 2.1. Ethics of Animal Experimentation

The use of experimental animals in this study was approved by the Institutional Animal Care and Use Committee at Chungbuk National University (Approval No. CBNUA-111R-18-01). All procedures involving animals and animal care were conducted in accordance with the guidelines issued by this committee.

### 2.2. Isolation and Culture of Rat Brain Cells

Each type of glial cell was isolated and cultured according to a previous method [14]. Briefly, we cryoanesthetized and decapitated postnatal day 1 (P1) Sprague-Dawley rats (DBL, Eumseong, Korea), and the meninges of the brain were removed in cold Hanks balanced salt solution containing D-glucose and sucrose (Gibco, Gaithersburg, MD, USA). The meninges-free cerebra were mechanically dissociated using a pipette until homogenous. Next, the dissociated cells were centrifuged for 10 min at 500 g, 4 °C. After discarding the supernatant, the settled cells were resuspended in DMEM20S medium (DMEM, 20% fetal bovine serum (FBS), 4 mM L-glutamine, 1 mM sodium pyruvate, 1% antibiotic-antimycotic (AA)). The cell suspension was plated and maintained in a 75T-flask coated with 0.01% poly-L-lysine for 7–10 days. When mixed glial cells reached a confluence of 80–90%, the flask was shaken on a shaking incubator for 1 h at 200 rpm, 37 °C. After discarding the media with microglia, 10 mL DMEM20S medium was added to the flask. The flask was then shaken in a shaking incubator for 18–20 h at 200 rpm, 37 °C to obtain OPCs. The media containing detached OPCs was plated in 6-well, 24-well, and 96-well plates coated with poly-L-lysine. Cells were then grown in OPC medium (DMEM, 4 mM L-glutamine, 1 mM sodium pyruvate, 0.1% bovine serum albumin, 50 μg/mL apo-transferrin, 5 μg/mL insulin, 30 nM sodium selenite, 10 nM D-biotin, 10 nM hydrocortisone, 10 ng/mL PDGF-AA, 10 ng/mL b-FGF) for 10–12 days at 37 °C in a 5% CO_2_ incubator. Astrocytes attached to the bottom of 75T-flask were separated by 0.25% trypsin-EDTA treatment. These cells were then plated in 6-well, 24-well, and 96-well plates and maintained in astrocyte medium (MEM, 20 mM glucose, 38 mM sodium bicarbonate, 1 mM sodium pyruvate, 5% FBS, 5% horse serum, 100 μM non-essential amino acid, 2 mM L-glutamine, 1% AA) for 7–10 days at 37 °C in a 5% CO_2_ incubator. Mature oligodendrocytes (mOLs) were obtained by differentiation of OPCs according to a previously published method [15]. Briefly, OPCs were maintained in mOL medium (DMEM, 4 mM L-glutamine, 1 mM sodium pyruvate, 0.1% bovine serum albumin, 50 µg/mL apo-transferrin, 5 µg/mL insulin, 30 nM sodium selenite, 10 nM D-biotin, 10 nM hydrocortisone, 40 ng/mL thyroxine, 40 ng/mL triidothyronine) for 7–9 days at 37 °C in a 5% CO_2_ incubator. Cortical neurons were isolated and cultured according to the previous method with a slight modification [16]. The meninges-free cerebra from neonate rats were chemically dissociated by 1 mL papain (2 mg/mL) and 200 μL DNase I (2.5 mg/mL), and mechanically dissociated using a pipette until homogenous. Next, the dissociated cells were centrifuged for 10 min at 500× *g*, 4 °C. After discarding the supernatant, the settled cells were resuspended in plating medium (DMEM, 10% FBS, 1% AA). The cell suspension was plated and maintained in a 75T-flask coated with poly-L-lysine. After 4 h, the medium was changed to neurobasal medium (Neurobasal-A medium, 10% B27 supplement, 1% AA) and maintained for 7–9 days at 37 °C in a 5% CO_2_ incubator.

### 2.3. Identification of Brain Cell Types

We used morphology and phenotypical antigenicity to identify different cell types—i.e., OPCs, mOLs, astrocytes, and cortical neurons. Nine days after starting the culture, the shapes of the cells were observed under a phase contrast microscope (CK40, Olympus, Tokyo, Japan). In addition, immunofluorescence staining was performed with specific markers for OPCs (anti-PDGFRα antibody), mOLs (anti-myelin basic protein (MBP) antibody), astrocytes (anti-glial fibrillary acidic protein (GFAP) antibody) and cortical neurons (anti-NeuN antibody).

### 2.4. Preparation of uf-UPs and Exposure to Cells

Working suspensions of uf-UPs were prepared according to the previous method [17]. Briefly, 2 mg of UPs (NIST1648A, Sigma, St. Louis, MO, USA) were suspended in 10 mL phosphate-buffered saline (PBS), vortexed for 1 min, sonicated for 45 min using an ultrasonicator, and filtered through a 0.2 μm syringe filter (Sartorius, Goettingen, Germany). To prepare various concentrations (2, 20, 200 μg/mL) of working suspensions, uf-UPs were resuspended in a serum-free culture medium. Each cell type was treated with the working suspension for 24 h at 37 °C in a 5% CO_2_ incubator. Hydrogen peroxide (100 μM) was used as a positive control.

### 2.5. Cell Viability Analysis

Cell viability was measured using MTT assays. MTT salts (10 μL of 5 mg/mL, Sigma, St. Louis, MO, USA) were added to cells exposed to uf-UPs in each well and cells were maintained for 4 h at 37 °C. Afterward, we added 110 μL solubilization buffer (27 mL isopropanol, 3 mL Triton X-100, 2.5 μL HCl) and each well was then incubated in a shaking incubator at room temperature for 10 min. Absorbance was measured at 570 nm by a spectrophotometer (Bio-Rad, Hercules, CA, USA) to determine optical density values.

### 2.6. Hoechst Staining

The proportion of dead cells was determined using Hoechst staining. Cells exposed to uf-UPs were fixed with 4% paraformaldehyde (PFA) in PBS for 20 min and washed with PBS twice for 5 min each. The cells were then incubated with 2 μg/mL Hoechst 33258 (Sigma, St. Louis, MO, USA) solution for 15 min at 37 °C followed by rinsing with PBS. Stained cells were observed under a multipurpose microscope with an epifluorescence attachment (DMLB, Leica, Wetzlar, Germany). Cells with condensed or fragmented nuclei after Hoechst staining were determined to be dead. Three to four wells were observed for each condition, and images were obtained from three areas (0.4 mm^2^/area) for each well.

### 2.7. Measurement of ROS Production

ROS production in cells was measured using dichlorofluorescein (DCF) assays and dihydroethidine (DHE) staining. DCF assays were performed according to a previously published method [18]. Briefly, cells exposed to uf-UPs were incubated in 100 μM 2′,7′-dichlorofluorescein diacetate for 30 min at 37 °C in a 5% CO_2_ incubator. Cell fluorescence measured using a SpectraMax M5e (Molecular Devices, San Jose, CA, USA). For DHE staining, cells exposed to uf-UPs were incubated with 10 μM DHE for 30 min at 37 °C in a 5% CO_2_ incubator. Stained cells were then observed under a multipurpose microscope with an epifluorescence attachment (DMLB, Leica, Wetzlar, Germany).

### 2.8. Measurement of Total Antioxidant Capacity

Total antioxidant capacities (TACs) were measured using cupric reducing antioxidant capacity (CUPRAC) assays as previously described [19]. Briefly, the lysates of the cells exposed to uf-UPs were diluted in a 0.25 mM bathocuproinedisulfonic acid disodium salt solution. Next, the diluted lysates were mixed with 0.5 mM CuSO4 and incubated for 3 min at room temperature, after which they were treated with 0.01 M EDTA to stop the reaction. Absorbance was measured at 492 nm, and TAC was expressed as copper reducing equivalents in μM.

### 2.9. Experimental Animals and Exposure of uf-UPs

Seven-week-old male Balb/c mice (20–22 g, *n* = 6) were obtained from a commercial source (DBL, Eumseong, Korea). Mice were housed under standard laboratory conditions under a 12 h light/dark cycle at 24–26 °C and were provided ad libitum access to a commercial diet and water. Mice were divided into two groups: control (*n* = 3) and UP exposure groups (UP, *n* = 3). Prior to uf-UPs exposure, mice were lightly anesthetized with isoflurane, and 20 μL of the uf-UP solution (0.4 mg/mL in PBS) were gently instilled into the nasal cavities using a micropipette in a supine position. Mice in the UP group were exposed twice a day at 12 h intervals for five consecutive days over 4 weeks. Mice in the control group received 20 μL of PBS instead of the uf-UP solution.

### 2.10. Tissue Preparation

Two days after final instillation, mice were deeply anesthetized with a mixture of ketamine hydrochloride (100 mg/kg, Yuhan Co., Seoul, Korea) and xylazine hydrochloride (10 mg/kg, Bayer Korea, Seoul, Korea). They were then transcardially perfused with precooled saline and 4% paraformaldehyde (PFA) in 0.1 M PBS (pH 7.4). The brains were removed from the heads and postfixed in 4% PFA in cold room air (4 °C) for 8 h. The brains were dehydrated in 30% sucrose in PBS overnight for cryoprotection. Brain tissues were then embedded in OCT compound (Leica, Wetzlar, Germany) and were rapidly frozen in 2-methyl butane (Junsei, Tokyo, Japan) adjusted to its freezing point with liquid nitrogen. Tissues were sectioned to a thickness of 50 μm using a cryostat (CM3050S, Leica, Wetzlar, Germany). Coronal slices were collected in PBS and transferred to six-well plates in a consecutive manner. A series of coronal sections was placed in each well at intervals of 300 μm.

### 2.11. Immunofluorescence Staining

Cells were fixed by 4% PFA in PBS for 15 min. To block nonspecific binding, cells and brain sections were incubated in PBS containing 10% normal goat or horse serum for 30 min. They were then incubated overnight at 4 °C with rat anti-platelet-derived growth factor receptor α (PDGFRα, 1:200, BD Bioscience, San Jose, CA, USA), rat anti-MBP (1:500, Millipore, Burlington, USA), mouse anti-GFAP (1:100, Biogenex, Fremont, CA, USA), and mouse anti-NeuN (1:100, Chemicon, Gaithersburg, MD, USA) antibodies, respectively. Next, the cells and sections were incubated for 2 h at room temperature with Cy2-labeled goat anti-rat IgG (1:500, Jackson ImmunoResearch Laboratories, West Grove, PA, USA) for anti-PDGFRα and MBP antibodies, and Cy2-labeled horse anti-mouse IgG (1:200, Vector, Burlingame, CA, USA) for the anti-GFAP and NeuN antibodies. Between each step, cells and sections were washed 3 times with PBS for 10 min. Finally, stained cells and sections were observed under a multipurpose microscope with an epifluorescence attachment (DMLB, Leica, Wetzlar, Germany).

### 2.12. Fluoro-Jade B Staining

The brain sections were incubated in 0.06% potassium permanganate solution for 10 min and subsequently in a 0.004% Fluoro-Jade B (FJB, Millipore, Burlington, MA, USA) solution containing 0.1% glacial acetic acid for 20 min at room temperature. Stained sections were then placed on a warm plate at 50 °C, and mounted with DPX, a non-aqueous non-fluorescent plastic mounting media (Sigma, St. Louis, USA). For FJB double staining, the sections were first immunostained with rabbit anti-NG2 (1:100, Millipore, Burlington, MA, USA) and rabbit anti-carbonic anhydrase-II (CA-II, 1:100, Abcam, Cambridge, USA) as primary antibodies overnight, and were then incubated with Cy3-labeled goat anti-rabbit IgG (1:500, Jackson ImmunoResarch Laboratories, West Grove, PA, USA) for 2 h. The immunostained sections were incubated in 0.06% potassium permanganate solution for 10 min and subsequently in 0.004% FJB solution for 20 min. The double stained sections were then dried and mounted with DPX. The number of FJB-positive dead cells was counted in two areas (100 μm^2^/area) randomly selected from the slides from three mice of each group.

### 2.13. Statistical Analysis

Data were expressed as mean ± standard error of the mean (SEM). Statistical differences were calculated by conducting Student’s t-tests and one-way analyses of variance, followed by Bonferroni post hoc tests. All analyses were performed using Prism 5 (GraphPad, San Diego, CA, USA). *p* values less than 0.05 were defined as statistically significant.

## 3. Results

### 3.1. Identification of Primary Brain Cells

**Figure 1:**
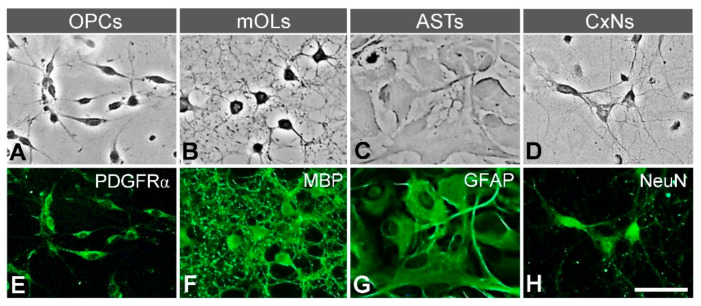
Identification of neural cells isolated from neonate rat brains. (**A**–**D**) Morphology visualized by phase contrast microscope. Each type—OPCs, mOLs, astrocytes (ASTs), and cortical neurons (CxNs) shows a characteristic shape. (**E**–**H**) Expression of specific antigens. OPCs, mOLs, ASTs, and CxNs are labeled with anti-PDGFRα, MBP, GFAP, and NeuN antibodies, respectively. Scale bar = 50 μm.

Phase contrast and immunofluorescent microscopy identified the typical morphology and specific antigen expression patterns of each cell type isolated from neonate rat brains. OPCs had oval cell bodies with bipolar processes, and most OPCs expressed PDGFRα—a specific marker of OPCs—in their cytoplasm (Figure 1A,E). Unlike OPCs, mOLs had round cell bodies with multipolar processes in a cobweb shape. Moreover, most mOLs expressed MBP, a specific marker for mOLs, in their cell bodies and processes (Figure 1B,F). The cell bodies of cultured astrocytes were larger than other types of brain cells, and all astrocytes specifically expressed GFAP (Figure 1C,G). The cell bodies of cortical neurons with long thin multipolar processes had a fusiform or pyramidal shape. Most cortical neurons were labeled with NeuN, a specific marker for neurons (Figure 1D,H). Taken together, the agreement of the morphological and antigen expression data suggests that the procedure for isolating each cell type was properly performed and accurate.

### 3.2. Cell Survival and Damage

**Figure 2:**
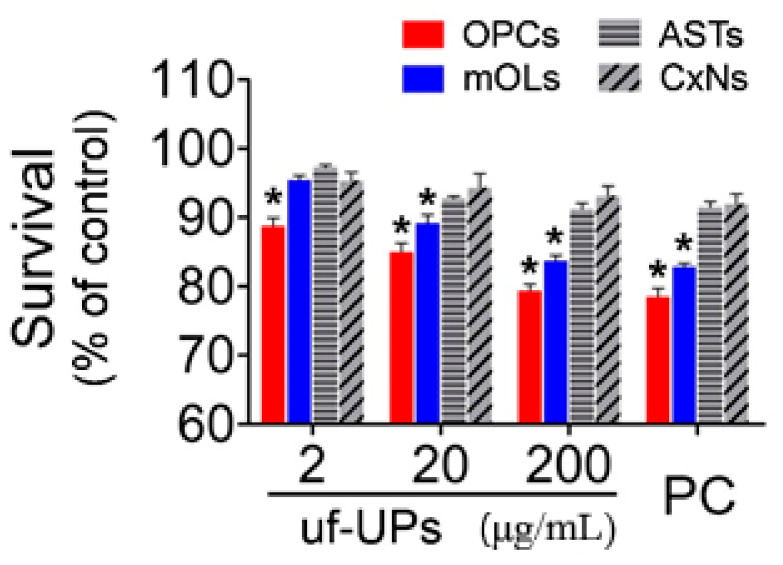
Cell survival analysis using MTT assay. Survival rates of OPCs and mOLs are significantly lower than those of ASTs and CxNs from 2 and 20 μg/mL uf-UPs, respectively. Hydrogen peroxide (H_2_O_2_, 100 μM) is used as a positive control (PC). Note that the survival rate of each cell type at 200 μg/mL uf-UPs is similar to the rate at 100 μM of hydrogen peroxide. Data are expressed as percentage of the control and represent mean ± SEM (*n* = 8). * *p* < 0.05 vs. ASTs and CxNs.

**Figure 3:**
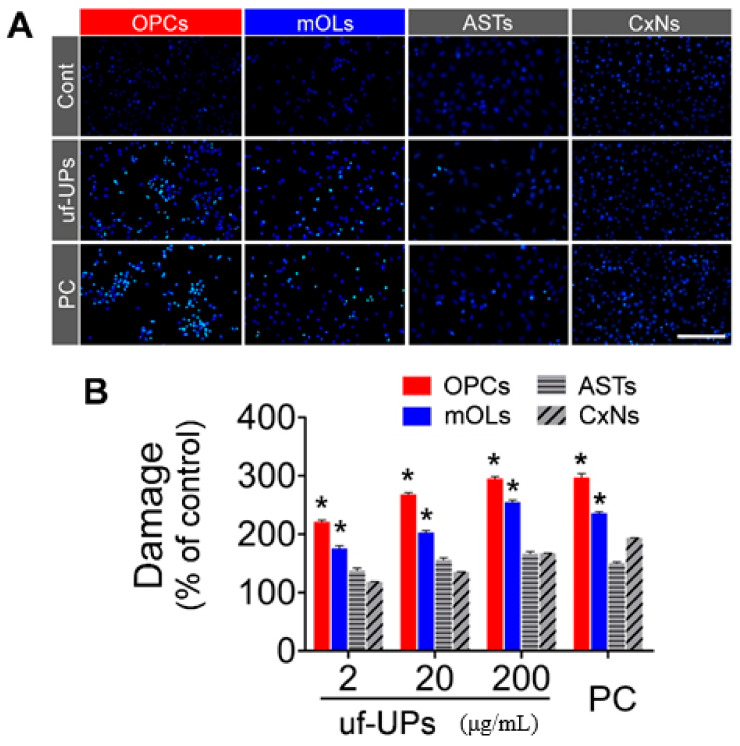
Cell damage analysis using Hoechst staining. (**A**) Hoechst staining. A large number of damaged OPCs and mOLs are visible after uf-UP exposure (200 μg/mL). (**B**) Comparison of damaged cells under various uf-UP concentrations. A significantly greater proportion of OPCs and mOLs than ASTs and CxNs are damaged by more than 2 μg/mL uf-UP exposure. Hydrogen peroxide (H_2_O_2_, 100 μM) is used as positive control (PC). Note that the damage rate of each cell type at 200 μg/mL uf-UPs is similar to the rate at 100 μM hydrogen peroxide. Data are expressed as percentage of the control and represent mean ± SEM (*n* = 8). * *p* < 0.05 vs. ASTs and CxNs. Scale bar = 200 μm.

To examine the viability of brain cells exposed to uf-UPs, we performed MTT assays and Hoechst staining. MTT assays showed that exposure to uf-UPs remarkedly decreased the survival of OPCs and mOLs in a dose-dependent manner (Figure 2). The survival rates of OPCs and mOLs were significantly lower than those of astrocytes and cortical neurons at concentrations greater than 2 and 20 μg/mL uf-UPs, respectively. Hoechst staining also showed that damaged OPCs and mOLs with condensed and fragmented nuclei were more abundant at 200 μg/mL uf-UPs (Figure 3A). Furthermore, cell counting analysis demonstrated that exposure to uf-UPs increased the number of damaged OPCs and mOLs in a dose-dependent manner (Figure 3B). The damage rates of OPCs and mOLs were significantly higher than those of astrocytes and cortical neurons at concentrations greater than 2 μg/mL uf-UPs. The MTT assay and Hoechst staining results clearly demonstrate that OPCs and mOLs are more susceptible to damage induced by uf-UP exposure than astrocytes and cortical neurons.

### 3.3. ROS Production

**Figure 4:**
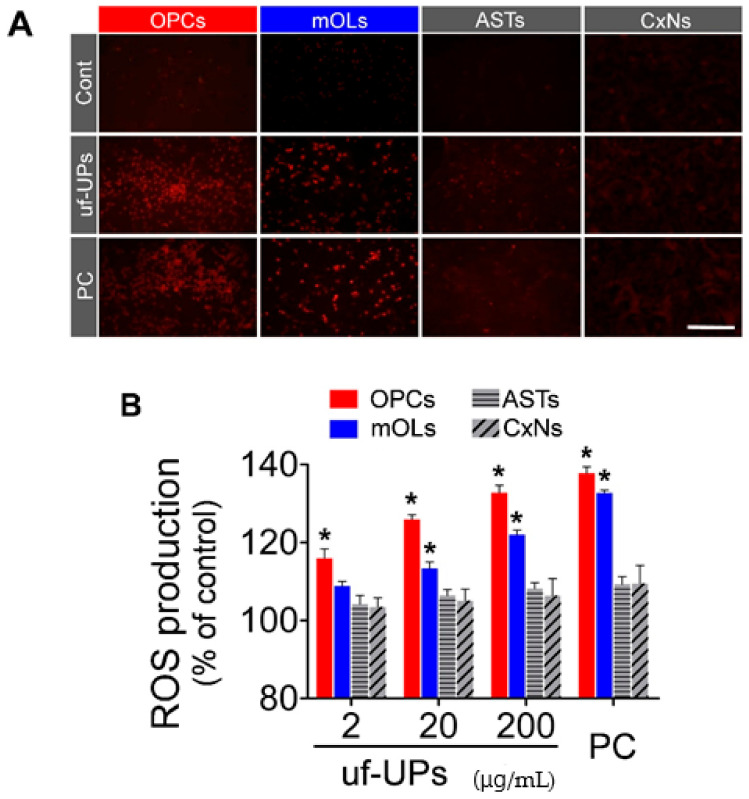
Reactive oxygen species (ROS) production analysis using DHE staining and DCF assays. (**A**) DHE staining. Large amounts of ROS are produced in OPCs and mOLs after uf-UP exposure (200 μg/mL). (**B**) DCF assay. The amounts of ROS produced by OPCs and mOLs are significantly higher the ROS levels produced by ASTs and CxNs after exposure to more than 2 and 20 μg/mL uf-UPs, respectively. Hydrogen peroxide (H_2_O_2_, 100 μM) is used as a positive control (PC). Note that ROS production in each cell type at 200 μg/mL uf-UPs is similar to the ROS production level at 100 μM hydrogen peroxide. Data are expressed as percentage of the control and represent mean ± SEM (*n* = 8). * *p* < 0.05 vs. ASTs and CxNs. Scale bar = 200 μm.

To measure ROS production in brain cells exposed to uf-UPs, we performed DHE staining and DCF assays. DHE staining showed that ROS production in OPCs and mOLs markedly increased when exposed to 200 μg/mL uf-UPs (Figure 4A). DCF assays also demonstrated that uf-UP exposure increased ROS production in OPCs and mOLs in a dose-dependent manner (Figure 4B). ROS production in OPCs and mOLs was significantly greater than in astrocytes and cortical neurons at concentrations greater than 2 and 20 μg/mL uf-UPs, respectively. Thus, the DHE staining and DCF assay results indicate that uf-UP exposure induces severe oxidative stress in OPCs and mOLs by increasing ROS production.

### 3.4. Total Antioxidant Capacity

**Figure 5:**
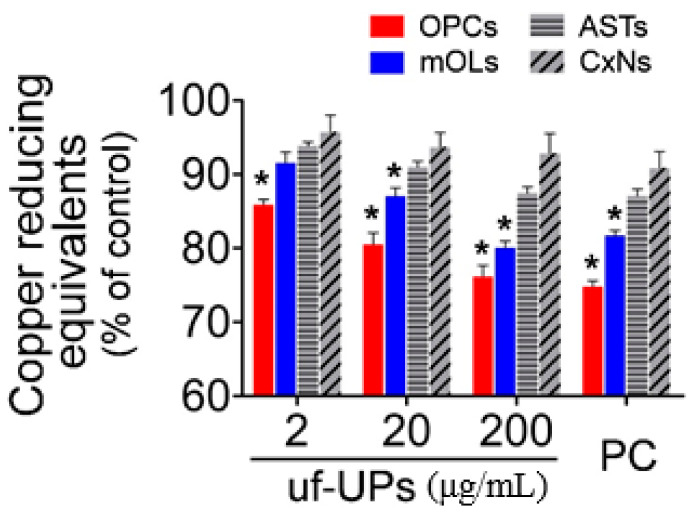
Total antioxidant capacity (TAC) assay. The TACs of OPCs and mOLs are significantly lower than those of ASTs and CxNs for more than 2 and 20 μg/mL uf-UP exposures, respectively. Hydrogen peroxide (H_2_O_2_, 100 μM) is used as a positive control (PC). Note that the TAC levels of each cell type at 200 μg/mL uf-UPs isare similar to the level at 100 μM of hydrogen peroxide. Data are expressed as a percentage of the control and represent mean ± SEM (*n* = 8). * *p* < 0.05 vs. ASTs and CxNs.

To evaluate the TACs of brain cells exposed to uf-UPs, we performed CUPRAC assays, which measure the capacity of antioxidants by Cu^2+^ reduction in the presence of bathocuproinedisulfonic acid disodium. Our results indicate that exposure to uf-UPs markedly decreased the TACs of OPCs and mOLs in a dose-dependent manner (Figure 5). Moreover, the TACs of OPCs and mOLs were significantly lower than those of astrocytes and cortical neurons at concentrations greater than 2 and 20 μg/mL uf-UPs, respectively. Taken together, these results indicate that reduced TACs inhibited the protection of OPCs and mOLs from enhanced oxidative stress induced by uf-UP exposure.

### 3.5. Oligodendrocyte Damage in Cerebellar White Matter

**Figure 6:**
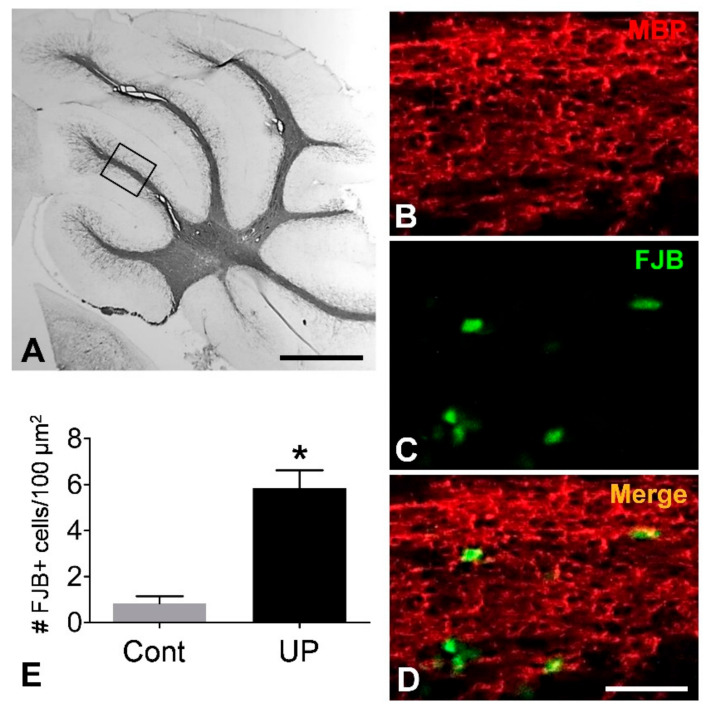
Detection of dead cells in the cerebellar white matter of mice exposed to uf-UPs for 4 weeks. Cerebellar white matter is labeled with an anti-MBP antibody (**A**). The white matter in the cerebellar folium (shown in the box in panel **A**) contains Fluoro-Jade B (FJB)-positive cells (**B**–**D**). The number of FJB-positive cells in the uf-UP exposure group (UP) is significantly higher than in the control group (**E**). * *p* < 0.05 vs. control. Scale bars in A = 500 μm, in **D** for **B**–**D** = 25 μm.

**Figure 7:**
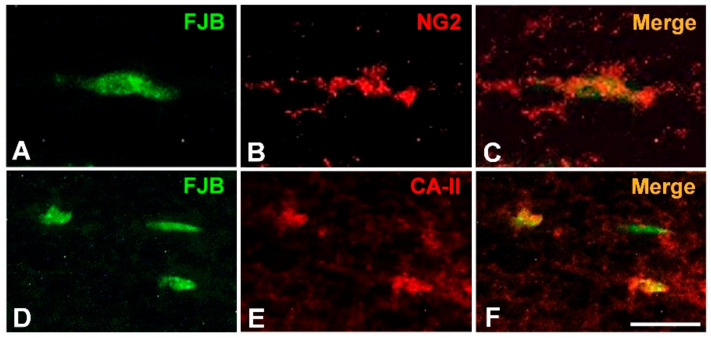
Identification of FJB-positive cells in the white matter of mice exposed to uf-UPs for 4 weeks. Most FJB-positive cells (green) in the white matter are double-labeled with either NG2 (red), an adult OPC marker (**A**–**C**), or CA-II (red), an mOL marker (**D**–**F**). Scale bar = 10 μm.

To examine if uf-UPs damage adult OPCs and mOLs *in vivo*, we performed FJB staining. In the sagittal section of the cerebellum, MBP-immunoreactivity was observed in the white matter of the folia and in the deep cerebellum (Figure 6A). In uf-UP-exposed mice (UP group), a considerable number of FJB-positive cells were detected in the MBP-positive white matter of the cerebellar folium (Figure 6B–D). Cell counting analysis clearly indicated that the number of FJB-positive cells within 100 μm^2^ sections of the UP group were significantly higher than in those of the control group (Figure 6E). Furthermore, double-labeling analysis demonstrated that FJB-positive cells overlapped with NG2-positive adult OPCs (Figure 7A–C) and CA-II-positive mOLs (Figure 7D–F) in cerebellar white matter. These results collectively demonstrate that four-week-long exposure to uf-UPs can damage OPCs and mOLs in the mouse brain.

## 4. Discussion

The hypothesis that air pollution affects brain health was raised a long time ago. Many previous studies that investigated the correlation between air pollutants and MS suggest a close linkage. In a study in Lombardy, PM10 was found to be closely related to higher recurrence rates in MS patients [9]. Another study in Atlanta (GA, USA) showed that MS prevalence tended to cluster within large metropolitan areas, suggesting that PM10 is a potential etiology of MS in women [20]. Similarly, prevalent MS cases between 2003–2013 also showed a clustered pattern in Tehran, Iran, revealing that long-term exposure to air pollutants including PM10, SO_2_, NO_2_, and NOx increases the risk of MS onset [8]. Air pollution seems to have a toxic impact on children as well as adults. PM2.5, along with other air pollutants including CO, SO_2_, and Pb, is implicated with pediatric MS and cognitive delay in children [21]. In addition, exposure to ultrafine PMs accompanying tobacco smoke has also been found to increase the incidence of MS in children [22]. Despite a consensus about the positive correlation between PMs and MS, to date there is little experimental evidence regarding the mechanism by which this link is affected. In the present study, we demonstrated that exposure to uf-UPs results in relatively more severe damage to OPCs and mOLs than to other brain cells such as astrocytes and cortical neurons. This was likely due to the fact that in these cell types, uf-UP exposure caused increased ROS production and concurrent decreases in TAC. Moreover, both pathologies were induced in a dose-dependent manner. These results are in accord with previous reports regarding the vulnerability of OPCs and mOLs to oxidative stress. For example, the lack of antioxidant enzymes such as glutathione has been shown to mean that OPCs are more susceptible than astrocytes to oxidative stress induced by exposure to low-energy blue light sources [13]. Similarly, mOLs can be easily damaged during the formation and maintenance of myelin, since large amounts of iron are needed as a cofactor in cholesterol biosynthesis of the myelin sheath. However, cytoplasmic iron in mOLs may produce highly reactive hydroxyl radicals (OH^•−^) through the Fenton reaction [23]. Thus, the high vulnerability of OPCs and mOLs to uf-UPs seems to result from excessive oxidative stress and poor protective mechanisms.

In the present study, we found that OPCs are more vulnerable to uf-UP-induced oxidative stress than mOLs. The majority of OPCs originates from the ventral ventricular zone during the late stages of brain development, and migrates throughout the brain [24]. Although most migrated OPCs differentiate into mOLs, some remain as adult OPCs, and account for approximately 5% of all brain cells [25]. Adult OPCs play a critical role in the restoration of function through remyelination after demyelination [11,12,26]. Despite being in the same cell lineage, OPCs and mOLs are quite different in morphology, function, proliferative capacity, and phenotypic antigenicity [10]. In addition, they may also differ in their protective mechanisms against oxidative stress, according to their maturation. Empirical work has suggested that premyelinating OLs (late OPCs) were more vulnerable than mOLs to the same degree of oxidative stress induced by glutathione depletion [27]. This suggests that late OPCs are more dependent on glutathione alone than mOLs when scavenging ROS. Recently, we found that OPCs are more easily damaged by hydrogen peroxide-induced oxidative stress than mOLs, since αB-crystallin, a chaperone protein is exclusively expressed in mOLs, but not in OPCs [28]. Furthermore, αB-crystallin expressed in mOLs activates the Akt signaling pathway, leading to greater survival rates than OPCs even under oxidative stress conditions [29]. Taken together, we hypothesize that uf-UPs primarily damage adult OPCs in the white matter, resulting in a reduced remyelination capacity. Subsequently, uf-UPs seem to cause damage to mOLs, which directly induces demyelination. This hypothesis agrees well with the results of our animal experiments, in which we detected both FJB-positive OPCs and mOLs in uf-UP-exposed white matter.

The underlying mechanism responsible for uf-UP-induced oxidative stress in both OPCs and mOLs remains unclear. However, there are several clues from previous investigations. Diesel exhaust particles, another standard reference material of the National Institute of Standard and Technology (NIST) have been found to activate NADPH oxidase, leading to increase superoxide (O2^•−^) production in isolated rat brain capillaries and mouse microglia [17,30]. In contrast, the inhibition of NADPH oxidase decreased the death rate of OLs after brain trauma [31]. These studies suggest that NADPH oxidase activation may be an important cause of the high vulnerability of OLs to oxidative stress induced by uf-UPs. UPs are a standard reference material of NIST, and are made up of various components collected from the atmosphere of a factory near St. Louis (MO, USA). According to the certificate of analysis provided by NIST, UPs contain polycyclic aromatic hydrocarbons (PAHs), nitro-substituted PAHs, polychlorinated biphenyl congeners, and chlorinated pesticides in atmospheric particulate material as well as in similar matrices. In particular, PAHs in UPs reportedly produce ROS by cytochrome P450 and dihydrodiol dehydrogenase and are also expected to have a direct toxic effect on OLs [32,33]. In addition, future studies should examine whether inhibition of ROS-producing enzymes activated by uf-UPs affect the survival of OLs.

Our in vitro and in vivo data strongly suggest that air pollution with ultrafine PMs is, at least, a partial cause of the onset and recurrence of MS in humans. Moreover, we speculate that our findings will provide a direction for the attenuation of clinical symptoms as well as treatment of MS. However, there are limitations in the methodological aspects of the present study. Unlike usual ultrafine PM exposure via breathing, we intranasally instilled uf-UP suspension (20 μL, 0.4 mg/mL) to mice twice a day at 12 h intervals for five consecutive days over four weeks. Differences in exposure ways disturbed conversion to the concentration in the air of exposed uf-UPs for comparison. In addition, considering long-term exposure under polluted atmosphere, four-week exposure was a relatively short period. Therefore, more studies are necessary to validate means of exposure and determine the concentration and exposure period of uf-UPs affecting the brain in the future.

In summary, we found that OPCs and mOLs were more severely damaged by exposure to uf-UPs than other brain cells in vitro, ostensibly due to dose-dependent increases in ROS production and decreases in TAC. Damage to adult OPCs and mOLs was also observed in the cerebellar white matter of mice exposed to uf-UPs for four weeks. Our findings suggest that oxidative stress caused by uf-UPs reduces remyelination capacity by causing the death of adult OPCs and induces demyelination by damaging mOLs.
